# Prevalence of attention deficit hyperactivity disorder among children and adolescents in Spain: a systematic review and meta-analysis of epidemiological studies

**DOI:** 10.1186/1471-244X-12-168

**Published:** 2012-10-12

**Authors:** Ferrán Catalá-López, Salvador Peiró, Manuel Ridao, Gabriel Sanfélix-Gimeno, Ricard Gènova-Maleras, Miguel A Catalá

**Affiliations:** 1 Centro Superior de Investigación en Salud Pública (CSISP), Valencia, Spain; 2 Fundación Instituto de Investigación en Servicios de Salud, Valencia, Spain; 3 Instituto Aragonés de Ciencias de la Salud (I+CS), Zaragoza, Spain; 4 Primary Care General Directorate, Regional Health Council, Madrid, Spain; 5 University of Valencia, Valencia, Spain

## Abstract

**Background:**

Attention deficit hyperactivity disorder (ADHD) is a commonly diagnosed neuropsychiatric disorder in childhood, but the frequency of the condition is not well established in many countries. The aim of the present study was to quantify the overall prevalence of ADHD among children and adolescents in Spain by means of a systematic review and meta-analysis.

**Methods:**

PubMed/MEDLINE, IME, IBECS and TESEO were comprehensively searched. Original reports were selected if they provided data on prevalence estimates of ADHD among people under 18 years old in Spain and were cross-sectional, observational epidemiological studies. Information from included studies was systematically extracted and evaluated. Overall pooled-prevalence estimates of ADHD were calculated using random-effects models. Sources of heterogeneity were explored by means sub-groups analyses and univariate meta-regressions.

**Results:**

Fourteen epidemiological studies (13,026 subjects) were selected. The overall pooled-prevalence of ADHD was estimated at 6.8% [95% confidence interval (CI) 4.9 – 8.8%] representing 361,580 (95% CI 260,550 – 467,927) children and adolescents in the community. There was significant heterogeneity (*P* < 0.001), which was incompletely explained by subgroup analyses and meta-regressions.

**Conclusions:**

Our findings suggest that the prevalence of ADHD among children and adolescents in Spain is consistent with previous studies conducted in other countries and regions. This study represents a first step in estimating the national burden of ADHD that will be essential to building evidence-based programs and services.

## Background

Attention deficit hyperactivity disorder (ADHD) can be defined as a condition starting in childhood, that comprises a persistent pattern of symptoms of hyperactivity, impulsiveness and/or lack of attention, more frequent and severe than usual for that age, and causing a significant impairment in school or work performance and in the activities of daily life. ADHD is a common neuropsychiatric disorder, with a high impact on the health system and the community in terms of economic costs, family stress, academic and vocational adversity and a clear negative effect on the self-esteem of the subject affected [1].

Currently, there exist two diagnostic criteria in regular use to diagnose ADHD in children and adolescents, DSM-IV and ICD-10. Both classifications utilise lists of behaviours to consider in the process of diagnosing hyperactive conditions. The main differences between DSM-IV and ICD-10 pertain to the concomitance of the three domains (inattention, hyperactivity and impulsivity), the exclusion of comorbidity and the degree of pervasiveness. The ICD-10 criteria require a full set of symptoms in all three domains, whereas the DSM-IV recognizes three subtypes of the disorder – the predominantly inattentive type, the predominantly hyperactive-impulsive type and the combined type.

Despite its relevance in terms of public health, the frequency of the disorder is not well established in many countries, including Spain. This information may be necessary to improve the design of future studies on aetiological factors and disease distribution in the population, evaluate the effectiveness and cost-effectiveness of various interventions or programmes and provide representative reference values for evidence-based health services planning. In recent decades, several observational studies have been performed in different population groups and geographic areas. Epidemiological studies in several countries have used questionnaires and scales based on symptoms as a criterion for ADHD. According to previous studies, in Spain the prevalence of ADHD would be 3-14% in children aged 8-15 years in Valencia [2,3], 4-6% in children aged 6-15 years in Seville [4] and 1% in children aged 6-8 years in Navarre [5]. Therefore, it would be relevant that the data provided in the scientific literature were analysed through integrated approaches which allow for establishing the extent of ADHD and its epidemiological characteristics for the whole children and adolescent population.

In this context, the objective of this study was to perform a systematic review of the studies performed in Spain on the prevalence of ADHD in children and adolescents and combine its results in an overall estimation through meta-analysis techniques.

## Methods

### Literature search

A systematic review was performed to document the availability of prevalence data for ADHD among children and adolescents in Spain. Methods were consistent with those recommended by the Meta-analysis of Observational Studies in Epidemiology (MOOSE) group [6]. A broad comprehensive search for original studies (published between January 1980 and August 2011) was conducted in the following electronic databases:

1. PubMed/MEDLINE (via the U.S. National Library of Medicine): The following terms or keywords were used: *"attention deficit disorder with hyperactivity" [MeSH Terms], ("attention" [All Fields] AND "deficit" [All Fields] AND "disorder" [All Fields] AND "hyperactivity" [All Fields]), "attention deficit disorder with hyperactivity" [All Fields], "adhd" [All Fields], "hyperkinesis" [MeSH Terms]*, combining them with *"epidemiologic studies" [MeSH]*, *"prevalence" [MeSH Terms]* and with the geographic filtre proposed by Valderas et al. [7] for identifying studies performed in the Spanish population and minimizing bias regarding the indexing of geographical items.

2. *Índice Médico Español* (IME) and *Índice Bibliográfico Español en Ciencias de la Salud* (IBECS): The preferred terms *“TDAH”, “trastorno por déficit de atención”, “hiperactividad”*, and “*hipercinético*” were used.

3. TESEO database (database of Spanish PhD theses): The preferred terms *“TDAH”, “trastorno por déficit de atención”, “hiperactividad”*, and “*trastorno hipercinético*” were used as descriptors.

The full list of terms used is shown in the Additional file 1: "Search Terms Used in the Bibliographic Review". Furthermore, complementary hand-searches reviewing the literature of extracted articles were carried out.

### Selection of studies

The primary end-point was the prevalence of ADHD among children and adolescents. By design, we used the investigator-reported definitions of ADHD patients provided in each single study. Of the references resulting after the bibliographic review, those referring to original publications of epidemiological observational studies meeting the following criteria were selected: cross-sectional design and studies reporting data for current (point/past month) or period prevalence of ADHD among people under 18 years old in Spain. For the purpose of the primary analyses, studies with any of the following criteria were excluded: studies with samples selected in a clinical setting, studies on adult population, lack of information on relevant study issues (not specifying sample size, number of cases, or the reference population), editorials and review articles. No date (year of publication) or language restrictions were established.

### Data extraction

Information about design and participants were extracted as recommended by PRISMA (Preferred Reporting Items for Systematic Reviews and Meta-Analyses) guidelines [8]. The PRISMA and MOOSE checklists are provided in the Web Appendix (Additional file 2: “PRISMA and MOOSE checklists). Data extraction from source documents was done independently by two investigators (one psychiatrist and one epidemiologist) and verified. Disagreements were resolved by consensus. The investigators used a specific form specifically designed to extract data of methodological and scientific quality. The following variables were collected: author and year of publication, author affiliation (e.g. university, primary care, or hospital), journal title, characteristics of the population (including sample size and age), geographic area, origin of the sample (e.g. school or population-based), some methodological issues (e.g. diagnostic criteria, assessment tools and number of stages of evaluation, clinical interview, impairment criterion and source of information) and the main results. The prevalence rate data (e.g. defined as the percentage of subjects with ADHD) were obtained from the selected studies. If these results were not directly provided and it was feasible, they were calculated from the case and population data provided in each single study.

### Data analysis

The overall pooled-prevalence was estimated by random-effects meta-analysis using the inverse variance method [9,10]. Heterogeneity was evaluated using the Cochran’s chi-squared test (Cochran’s *Q*) and the *I*^*2*^ statistics [11,12]. Cochran's *Q* is the sum of the squared differences between each study's effect estimate and the overall effect estimate, weighted for the information provided by the particular study. *I*^*2*^ is the proportion of total variation observed between the studies attributable to differences between studies rather than sampling error. To investigate sources of heterogeneity, subgroups (from the characteristics of the population and study design) and univariate meta-regression analyses were defined. Particularly, because the large time span of the eligible studies, we explored trends over time using random-effects meta-regression with the year of publication as the explanatory variable. Similarly, we explored trends of prevalence variation with gender in terms of the male-to-female ratio. Because only a few covariates were individually significant, multivariate meta-regression or hierarchical models were not developed.

A sensitivity analysis was also conducted to examine the possible influence of single studies by excluding possible outlier (extreme) observations. The identification of a study as an outlier was not based on an a priori statistical criterion, but rather on visual evaluation of forest plot with all selected studies. Furthermore, to explore investigator-reported definitions of ADHD across the studies, we conducted sensitivity analyses to determine the robustness of effect size by excluding studies on the basis of the clinical ascertainment (e.g. consideration only of those studies including cases clinically confirmed which applied DSM criteria and/or those studies scoring above 1.5 standard deviation on different specific questionnaires).

We assessed publication bias using the funnel plot method.

All the analyses were performed using STATA 11 (StataCorp, College Station, TX, USA).

## Results

### Identification and selection of articles

**Figure 1:**
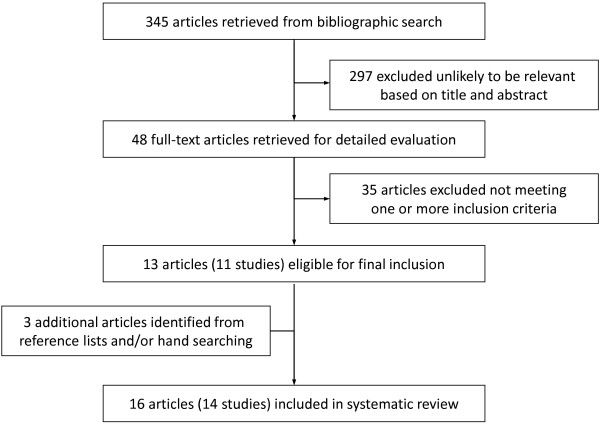
Flow diagram showing selection process of articles included in the systematic review.

Our initial searches yielded 345 literature references. After screening titles and abstracts, 48 articles were potentially eligible and were retrieved in full text. After a careful reading of these articles, 11 studies were found to meet the inclusion criteria. Complementary hand searches allowed for identifying 3 additional studies. Therefore, a total of 14 studies were included [2-5,13-24]. We excluded 35 reports (the reasons for exclusion are given in Additional file 3: “List of Excluded References and Reasons for Exclusion”). Figure 1 shows a flow diagram for the selection process of studies included in the systematic review.

### Characteristics of the studies

The 14 studies included in the systematic review and in the meta-analysis included a total of 13,026 children and adolescents. Table 1 shows the summary characteristics of the studies selected. The first author of most studies worked in an academic setting. With regard to the year of publication, half of the studies were published in the 90s [2-4,13-15,22,23]. Only three of the studies were performed in the general population [2,3,15,23] while in the rest the sample of children and adolescents was obtained from the school population [4,13,14,16-21]. Half of the studies were described through a two-stage design [2-4,15,16,18,20,22]: first, a psychometric screening that evaluated the presence of ADHD symptoms in children and adolescents; and second a clinical confirmation using standardised diagnostic criteria. The specfic characteristics in each study included in the systematic review are given in Table 2. Most of the studies using diagnostic criteria [2-4,13-16,20-23] applied the DSM-III-R and/or DSM-IV criteria of the American Psychiatric Association. In twelve studies [2-5,13,16,18-21,23] the prevalence of ADHD was calculated with information from at least 2 informers (in nine of them from parents and teachers), while in two studies [14,17] there was a single informer (the teachers) to assess the presence of the disorder. The fourteen studies provided the exact number of ADHD cases in the study population and the relevant prevalence rates, with values ranging from 1% to 14% [2,5,14]. Ten studies reported the male-to-female ratio [3,13-17,19-24] and the prevalence of ADHD was generally higher in men than in women, with a 4:1 ratio in four studies [14,17,22,24] and 2:1 in three studies [3,13,20].

**Table 1:** Summary characteristics of the 14 studies included in the systematic review

**Characteristic**	**Number (%)**
**Year of publication**	
1980-1989	2 (14.3)
1990-1999	7 (50.0)
2000-2011	5 (35.7)
**Journal title (abbreviated)**	
Rev Neurol	3 (21.4)
Acta Psychiatr Scand	1 (7.1)
Soc Psychiatry Psychiatr Epidemiol	1 (7.1)
Rev Psiquiat y Psicol Med	1 (7.1)
An Psiquiatr	1 (7.1)
Actas Luso Esp Neurol Psiquiatr Cienc Afines	1 (7.1)
Acta Pediatr Esp	1 (7.1)
Rev Pediatr Aten Primaria	1 (7.1)
Arch Pediatr	1 (7.1)
Pediatr Catalana	1 (7.1)
None/unpublished (e.g. PhD thesis)	3 (21.4)
**Peer reviewed journal**	
Yes	10 (71.4)
No	4 (28.6)
**Author affiliation**	
University	7 (50.0)
Primary care	3 (21.4)
Hospital	2 (14.3)
Other/non explicit	2 (14.3)
**Population age**	
Children and adolescents (under 17 years)	6 (42.9)
Children (under 12 years)	8 (57.1)
**Origin of sample**	
School	11 (78.6)
General population	3 (21.4)
**Reference to a diagnostic criterion**	
DSM-III-R	6 (42.9)
DSM-IV	4 (28.6)
Other/non explicit	4 (28.6)
**Impairment criterion**	
Yes	7 (50.0)
No	7 (50.0)
**Number of stages of evaluation**	
One	7 (50.0)
Two	7 (50.0)
**Inclusion of clinical interview**	
Yes	8 (57.1)
No	6 (42.9)
**Source of information**	
Parents and teachers	9 (64.3)
Parents and subjects	2 (14.3)
Teachers	2 (14.3)
Parents, teachers and subjects	1 (7.1)

**Table 2:** Characteristics of the studies included in the systematic review on knowledge on ADHD prevalence among children and adolescents in Spain

**Author, year of publication**	**Region or county**	**Study population**	**Age (in years)**	**Origin of sample (size)**	**Assessment tools**	**Clinical interview**	**Source of information**	**Reference to a diagnostic criterion**	**Impairment criterion**	**Prevalence estimate (%)**
					**Response rate (%)**					**Male-to-female ratio**
	**Geographic location**									
Guimón et al, 1980*	Biscay	Children	5-11.5	School ( N= 140 )	Hyperkinesia scales, perinatal history, neurological examination and Bender-Gestalt test, CAT, Corman test, PFT, intelligence scales (WISC, Terman-Merrill), academic performance	No	Parents and teachers	None or non explicit	Yes	8.0%
	North									4:1
					Non explicit					
Farré and Narbona, 1989*	Navarre	Children (only boys)	6-8	School ( N= 561 )	Conner’s scales, Raven's Colored Progressive Matrices, academic performance	No	Parents and teachers	None or non explicit	No	1.0%
	Northeast									
										-
					93%					
Gutiérrez Bengoechea, 1992	Asturias	Children	6-11	School ( N= 1,048 )	Conner’s scales, CBCL	Yes	Parents and teachers	DSM-III-R	No	4.5%
					>90%					4:1
	North									
Benjumea and Mojarro, 1993*	Seville	Children and adolescents	6-15	School ( N= 1,791 )	Conner’s scales, PACS	Yes	Parents and teachers	DSM-III-R	No	4.0%-6.0%
										-
					>60%					
	South									
Verdeguer, 1994	Castellon	Children	10	General population ( N= 325 )	Conner’s scales, Werry-Weiss-Peters activity rating scale, K-SADS-E, Raven's Colored Progressive Matrices, GAF scale, ADHD rating scale	Yes	Parents and teachers	DSM-III-R	Yes	7.1%
	East									
										10:1
					93%					
Gómez-Beneyto et al, 1994*	Valencia	Children and adolescents	8, 11 and 15	General population ( N = 1,127 )	CBCL, K-SADS-E, Raven's Colored Progressive Matrices, GAF scale	Yes	Parents and subjects	DSM-III-R	Yes	14.4%, 5.3%, 3.0%
	East									
										1.2-1.7:1
					94%					
Andrés-Carrasco et al, 1995 and 1999*	Valencia	Children	10	General population ( N = 387 )	K-SADS-E, Raven's Colored Progressive Matrices, GAF scale	Yes	Parents and subjects	DSM-III-R	Yes	8.0%
	East									2:1
					98%					
Eddy, 1997*	Barcelona	Children	7 and 8	School ( N= 263 )	Conner’s scales	No	Parents and teachers	DSM-III-R	No	5.7%-9.8%
					Non explicit					2:1
	Northeast									
Ruiz et al, 1999	Barcelona	Children	6-10	School ( N = 1,433 )	SNAP modified, VADTRS	No	Teachers	DSM-IV	Yes	14.0%
										4:1
	Northeast				98%					
García-Jiménez et al, 2005*	Navarre	Children and adolescents	6-12	School ( N= 222 )	Conner’s scales, Conner’s modified, ADHD rating scale	Yes	Parents, teachers and subjects	DSM-IV	Yes	9.0%
	Northeast									5.6:1
					82%					
Blázquez Almeria et al, 2005*	Barcelona	Children and adolescents	6-13	School ( N = 2,401 )	Conner’s modified, ADHD rating scale	No	Teachers	None or non explicit	No	12.2%
	Northeast									
					Non explicit					4:1
Rodríguez Hernández, 2006	Canary Islands	Children	7-10	School ( N= 595 )	SDQ	No	Parents and teachers	None or non explicit	No	3.9%
					89%					-
	South									
Rodríguez Molinero et al, 2009*	Castile- Leon	Children and adolescents	6-16	School ( N= 1,095 )	ADHD rating scale, Vanderbilt ADHD assessment scale, CSI	Yes	Parents and teachers	DSM-IV	Yes	6.7%
										2.3:1
	North									
					Non explicit					
Cardo et al, 2007 and 2011*	Majorca	Children and adolescents	6-12	School ( N= 1,509 )	ADHD rating scale	No	Parents and teachers	DSM-IV	No	1.2%-4.6%
										1:1.5
					Non explicit					
	East									

CAT: Children's Apperception Test; CBCL: Child Behavior Checklist; CSI: Child Symptom Inventory; GAF: Global Assessment of Functioning; K-SADS-E: Kiddy Schedule for affective diseases and Schizophrenia (epidemiological version); PACS: Parenteral account of children's symptoms; PFT: Rosenzweig Picture Frustration Test; SDQ: Strengths and Difficulties Questionnaires; SNAP-IV: Swanson, Nolan y Pelham, 4^th^ Edition; VADTRS: Vanderbilt ADHD Teacher Rating Scale.; WISC: Wechsler Intelligence Scale for Children.

*Published in a peer-reviewed journal.

### Overall meta-analysis and publication bias

**Figure 2:**
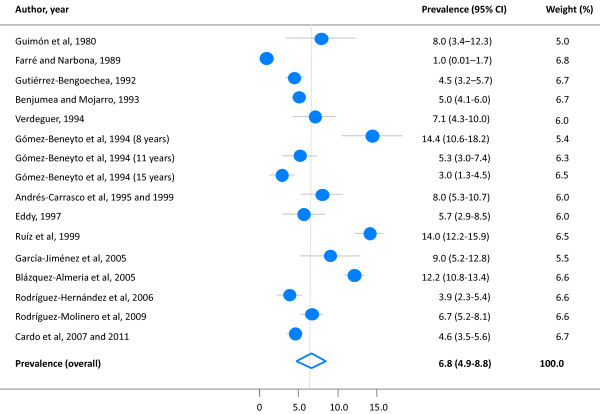
**Prevalence of ADHD among children and adolescents in Spain: meta-analysis.** Note: Random effects model. Cochran’s Q: χ^2^ = 367.8 (d.f. = 15), p < 0.001; I ^2^= 95.9%.

The forest plot in Figure 2 shows the data from the single studies and on the overall pooled-prevalence of ADHD from the baseline meta-analysis. Using the random effect model, an overall pooled-prevalence of ADHD of 6.8% (95% CI 4.9 – 8.8%) was obtained for children and adolescents with substantial between-study heterogeneity (*I*^*2*^ = 95.9%; *Q* statistic *P* < 0.001). From the projections of the current population [25] and the results of the baseline meta-analysis, it was estimated that in Spain ADHD would currently affect around 361,580 (95% CI 260,550 – 467,927) children and adolescents in the community.

**Figure 3:**
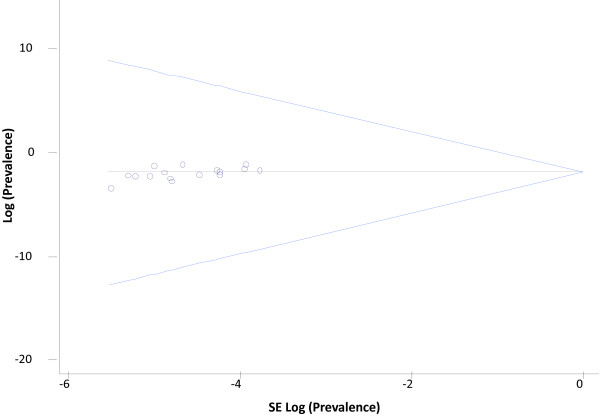
Publication bias: funnel plot.

Visual inspection of the funnel plot denoted no evidence of publication bias (Figure 3).

### Subgroup and univariate meta-regression analyses

**Figure 4:**
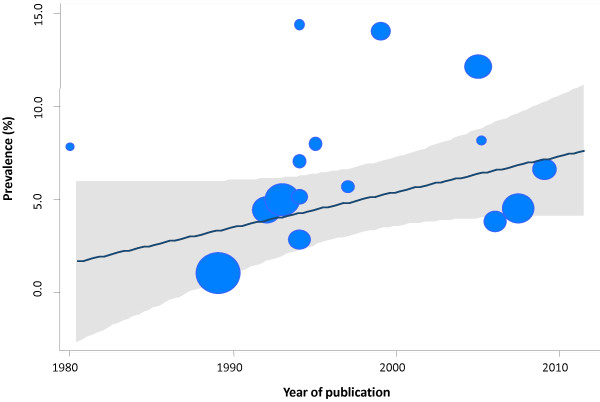
**Relationship between year of publication and the prevalences of ADHD among children and adolescents in Spain.** Meta-regression analysis. Note: The size of the bubble is inversely related to the variance of the study. The solid line represents the linear regression (year of publication as the meta-independent variable). The shaded area corresponds to the confidence intervals of the prediction.

**Figure 5:**
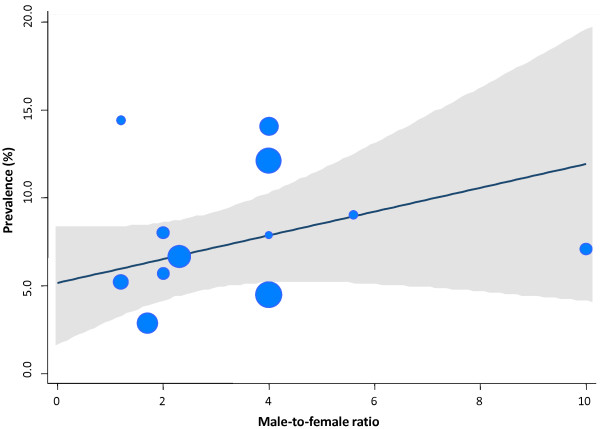
**Relationship between male-to-female ratio and the prevalences of ADHD among children and adolescents in Spain.** Meta-regression analysis. Note: The size of the bubble is inversely related to the variance of the study. The solid line represents the linear regression (male-to-female ratio as the meta-independent variable). The shaded area corresponds to the confidence intervals of the prediction.

It must be noted that there was a high degree of heterogeneity between the studies included in the review in terms of the prevalence calculated (*Q* = 367.8; *P* < 0.001). Therefore, it was considered important to investigate and try to explain the possible sources of heterogeneity that could be present in the studies included in the review. For this, subgroup analyses and univariate meta-regression analyses were performed. Table 3 shows the results of the meta-analysis by subgroups with the *I*^*2*^ indices. The variables “geographic area” and “among informers only teachers are included” would allow for explaining very partially part of the heterogeneity found. The results of random-effects meta-regression analyses that assessed the relationship between selected covariates and the observed prevalences in each single study is presented in Figures 4 and 5. There was a non statistically significant linear trend to explain effect size variation by year of publication (*P* = 0.537). Similarly, there was a non statistically significant linear trend to explain effect size variation by gender in terms of the male-to-female ratio (*P* = 0.557).

**Table 3:** Prevalence of ADHD among children and adolescents in Spain: subgroup meta-analysis and heterogeneity analysis

**Characteristics**	Observations* (N)	**Prevalence (%)**	**95% CI**	**I**^**2**^	**P value**
**Origin of sample**					
School	11	6.7	4.2-9.1	97.0%	< 0.001
General population	5	7.2	4.0-10.5	88.8%	< 0.001
**Geographic location**					
North/Northeast	8	7.6	3.8-11.4	97.9%	< 0.001
South	2	4.6	3.5-5.7	38.4%	0.203
East	6	6.6	4.3-8.9	86.7%	< 0.001
**Sample size**					
< 600 subjects	9	6.1	3.7-8.4	91.9%	< 0.001
> 600 subjects	7	7.7	5.0-10.4	96.4%	< 0.001
**Population age**					
Children (under 12 years)	10	7.0	4.1-9.9	96.0%	< 0.001
Children and adolescent (under 17 years)	6	6.6	3.9-9.3	95.6%	< 0.001
**Peer reviewed journal**					
Yes	12	6.7	4.4-8.9	95.9%	< 0.001
No	4	7.3	2.7-12.0	96.9%	< 0.001
**Reference to a diagnostic criterion**					
DSM-III-R	8	6.1	4.5-7.7	81.4%	< 0.001
DSM-IV	3	9.9	4.6-15.2	94.8%	< 0.001
None or not explicit	5	5.8	1.5-10.0	98.1%	< 0.001
**Impairment criterion**					
Yes	9	8.3	5.5-11.0	92.0%	< 0.001
No	7	5.2	2.5-7.9	97.2%	< 0.001
**Clinical interview**					
Yes	9	6.4	4.9-7.9	82.2%	< 0.001
No	7	7.0	3.0-10.9	98.1%	< 0.001
**Number of stages of evaluation**					
One	7	7.5	3.3-11.7	98.1%	< 0.001
Two	9	6.0	4.6-7.4	82.8%	< 0.001
**Number of informants**					
One	2	13.0	11.1-14.9	65.0%	0.091
Two	13	5.5	4.1-6.9	90.7%	< 0.001
Three	1	9.0	5.2-12.8	-	-
**Children are among the informants**					
Yes	5	7.6	4.1-11.2	89.4%	< 0.001
No	11	6.5	4.1-8.9	97.0%	< 0.001
**Teachers are the sole informants**					
Yes	2	13.0	11.1-14.9	65.0%	0.091
No	14	5.7	4.3-7.1	90.4%	< 0.001

*Note: Data set correspond to individual observations (n=16) because the study by Gómez-Beneyto contributed to analyses with three estimates (for people of 8, 11 and 15 year-old each).

### Sensitivity analyses

The results of the sensitivity analysis are provided in Additional file 4: “Uncertainty and sensitivity analyses”). The prevalence estimate after excluding the four estimations with extreme outliers [2,5,15,23] was consistent with other previous studies [26]. The overall pooled-prevalence of ADHD was 5.3% (95% CI 4.5 – 6.2%), with moderate between-study heterogeneity (*I*^*2*^ = 60.8%; Q statistic *P* = 0.003). Complementary sensitivity analyses based on the clinical ascertainment (e.g. consideration only studies including cases clinically confirmed by DSM criteria) and the choice of the statistical model did not make any noticeable difference for the above analyses (please see webappendix, Additional file 4). Particularly, in 7 out of 14 studies (6,175 children and adolescents) with clinically confirmed ADHD cases, the pooled-prevalence was 6.4% (95% CI 4.9 – 7.9%) with substantial between-study heterogeneity (*I*^*2*^ = 82.2%; Q statistic *P* < 0.001).

## Discussion

This study reviewed 14 epidemiological observational studies that analyse the prevalence of ADHD in children and adolescents in Spain, over more than 13,000 people. The main result was the identification of the epidemiological information for providing an estimation of the overall prevalence of disorder in the country. It also provides a greater precision than that resulting of the studies individually considered. Specifically, the results of the meta-analysis show a pooled-prevalence of ADHD of 6.8% in children and adolescents. These values would be generally consistent with those found in the European Union [26,27]. Wittchen et al. [27] recently estimated that 3.3 million children and adolescents aged 6-17 years have ADHD in the European Union, with a prevalence of 5%. The results of the meta-analysis by Polanczyk et al. [26] yielded a world prevalence equivalent to 5.3%, with similar values for developed regions.

ADHD is a major cause of personal impairment to patients and their families and place a substantial burden on the healthcare services [1]. Given the large amount of literature on ADHD in recent years, healthcare professionals and decision-makers must have up-to-date epidemiological information gathering the best scientific evidence that is useful for service planning and public health policy. Particularly, this information may be necessary to improve the design of future nationwide epidemiological studies, evaluate the impact of interventions or programmes on ADHD. With this regard, the prevalence measures provided can be used to further assess the non-fatal consequences of ADHD integrating this information in summary measures of population health, such as disability-adjusted life years [28]. Furthermore, where scientific evidence exists on the (cost-) effectiveness of particular health services and treatments, our results can also provide data inputs necessary for models of health impact assessments considering the implementation of alternative interventions.

This study has some limitations that must be considered. On the one hand, it shows the generic limitations of any systematic review and meta-analysis, particularly that its quality depends on the studies included and that, although the variability is controlled statistically by random effect models, strata are weighed by their characteristics and heterogeneity is measured, it must be always considered that the meta-analysis is a combination of sometimes disagreeing results. With this regard, the prevalence of ADHD ranged from 1% to 14% in the studies reviewed. Specifically, the study by Farré and Narbona [5] reported the lowest frequencies (1%). These values are probably influenced by the definition of a high critical score as hyperactivity index (> 18 of 30 points in the hyperactivity index of the Conners’ scales). In the same study, the authors recognised that, considering subjects with a critical score in at least one of the two questionnaires, the prevalence of ADHD was 6.4% (2.1% in parents, 3.4% in teachers and 0.8% both). Furthermore, in two studies [14,17] using a single informer (the teacher) the prevalence rates were higher (12-14%). This is consistent with the information from other authors reporting that using two informers instead of one usually provides a lower prevalence [26]. Along this, previous studies [29,30] found that teacher and parents agreement on questionnaires is often low, with teachers reporting more symptoms than parents. Both the DSM-IV [31] and the ICD-10 criteria [32] require that the main symptoms (attention deficit, hyperactivity and impulsiveness) occur in more than one setting (e.g., home and school). For example, Rojo et al. [33] analyzed the risk of ADHD characteristics among obese adolescents using a self-administered report of the Strengths and Difficulties Questionnaire (SDQ). As they mentioned, the sensitivity for predicting ADHD conditions was reported to be very low because they were based only on data from one informant (adolescents) and a clinical evaluation of the probable cases was not conducted. Although the aim of that study was not to establish prevalence estimates of ADHD, the authors provided a proportion of ADHD characteristics equals to 21.4% of adolescents aged 13-15 years. It is noteworthy that these results diverge somewhat from those obtained by Rodríguez-Hernández [18] and included in our meta-analysis (3.9% of children aged 7-10 years using parents/teachers’ SDQ).

As with other systematic reviews and meta-analyses [26,34] a significant heterogeneity was found in the prevalence measures, that were explained incompletely by analysis of subgroups and univariate meta-regressions. In our study, the variables “geographic area” (e.g. South region), “among informers only teachers are included” and/or “one informant” would allow for explaining very partially part of the heterogeneity found. Meta-regression on all epidemiological studies showed only a non statistically linear trend to explain effect variation by year of publication and male-to-female ratio, perhaps because of the limited power of our analysis (e.g. number of observations). These findings also suggest that other unknown factors could be important in accounting for between-study variations. For example, it is noteworthy that none of the studies included in our systematic review has studied the overall prevalence within the national population. The lack of whole population studies has been criticised in the past because selected populations (e.g. subnational/local different samples) and settings (e.g. population-based versus school-based studies) might introduce bias and some degree of uncertainty to the estimates. Previously, the revision by Skounti et al. [34] suggested that the characteristics of the population, the study methods and the diagnostic criterion differences could explain part of the changes in the ADHD prevalence rates.

The existence of nonindexed epidemiological studies in the databases consulted may have involved the loss of some locally relevant studies despite the extensive data searches we made (e.g. PubMed/MEDLINE, IME, IBECS and TESEO). Although an attempt was made to minimize this possible screening bias with specific searches in national databases and thesis dissertations, there may be other studies which have not been identified. However, publication bias is not anticipated (as denoted by funnel plot) because of we obtained a substantial proportion of data from unpublished studies. We also conducted subgroup and sensitivity analyses to assess uncertainty assumptions on the pooled ADHD-prevalence for study characteristics. Such an approach is important in assessing the validity of the assumptions made for the statistical calculations in meta-analyses. Unfortunately information on methodological quality of selected studies was insufficient to allow a detailed analysis of their quality.

The diagnosis of ADHD is complex and should be based on the clinical assessment confirmed by an expert on the recognition and treatment of it. In epidemiological studies there is no agreement about the instruments to be used for evaluating children with potential ADHD; there is also some controversy about the criteria to be used for defining a so-called “case”. These difficulties in the detection, diagnostic process and methods affect the epidemiological studies performed, originating changes that can lead to under- or over-diagnosing ADHD. In our meta-analysis, although based on a small subgroup of studies, the inclusion of those epidemiological studies that were restricted to clinically confirmed ADHD cases (e.g. DSM diagnostic criteria) led to a reduction of the pooled-prevalence from 6.8% (95% CI 4.9 – 8.8%) to 6.4% (95% CI 4.9 – 7.9%), which is even more consistent with the prevalence rates worldwide [26]. Similarly, people who screen negative do not undergo the clinical ascertainment by the specialists, therefore false negatives might have occurred in some epidemiological studies. In this revision, a number of studies did not mention any reference diagnostic criterion [5,17,18,24]. In addition, some used only screening scales with a low sensitivity and specificity and that are not valid as a single measurement for the diagnosis. Actually, DSM-IV and ICD-10 are the diagnostic criteria most commonly used. Both classifications describe the clinical condition of hyperactive children (ADHD/Hyperkinetic disorder) and use similar operative criteria for diagnosing it. However, as the ICD-10 diagnostic criteria are more restrictive, the diagnoses according to this classification will correspond to the most severe cases of ADHD according to DSM-IV criteria. Therefore, the prevalence studies taking ICD-10 as reference will probably yield lower rates than those using DSM-IV. ICD-10 and DSM-IV are also different when considering the subtypes of disorder.

The disagreement between the different studies in terms of case definition criteria involves the need for performing separate analyses for each criteria used for the diagnosis of ADHD. In our revision, no study reported expressly ICD-10 reference diagnoses. There exist evidences [35-37] that the prevalence of ADHD as defined in the DSM-IV can be somewhat higher than when defined according to DSM-III-R criteria, due to the inclusion of the types “with hyperactive-impulsive predominance” and “with attention deficit predominance” (that had been diagnosed as ADHD not specified in the DSM-III-R) [38]. For the diagnosis of ADHD, the DSM-III-R requires the presence of at least 8 symptoms of a total of 14; it does not include the requirement that they must occur in at least two settings and does not give a division into subtypes, and the severity criterion is based on the number of symptoms. In our subgroup meta-analysis, we confirmed that the use of DSM-IV vs DSM-III-R increases the prevalence rate. However, it is note worthy that the analyses of subgroups involve a loss of statistical power and, therefore, of precision and the unfeasibility to analyse population subgroups that would have been interesting (e.g. analysis by age or social class). As with other revisions [26,34] in virtually all the studies reviewed, regardless of the methods used, the prevalence rates of ADHD were significantly higher in men than in women. On the contrary, in the study by Cardo et al. [19] ADHD prevalence rates were slightly higher among women. This could be due to the fact that retained students, children with special educational needs and those with some known psychopathological diagnosis were excluded from the study, which would have underestimated prevalence in males.

## Conclusion

The prevalence of ADHD based on *evidence synthesis techniques* (systematic reviews and metanalysis) are readily calculated and useful for measuring the frequency of the disorder for a specific country. Particularly, our findings suggest that the prevalence of ADHD among children and adolescents is considerable in Spain. Our estimates are consistent with those previously reported in other countries and regions. Finally, this study also represents a first step in estimating the national burden of ADHD that will be essential to building evidence-based programs and services.

## Competing interests

The authors declare that they have no competing interests.

## Authors’ contributions

FCL conceived the study aims and design, and developed the study in discussions with MAC, SP, MR, GSG and RGM. FCL performed the analysis and drafted the initial manuscript. All authors contributed to interpretation of results, revised and commented on the manuscript for important intellectual content. All authors read and approved the final manuscript. FCL is guarantor of the manuscript.

## Pre-publication history

The pre-publication history for this paper can be accessed here:

http://www.biomedcentral.com/1471-244X/12/168/prepub

## Supplementary Material

Additional file 1 “Search Terms Used In The Bibliographic Review”. Click here for file

Additional file 2 PRISMA Checklist. MOOSE Checklist. Click here for file

Additional file 3 “List Of Excluded References And Reasons for Exclusion”. Click here for file

Additional file 4 “Uncertainty And Sensitivity Analyses”. Click here for file
