# Supplementary material for: Prevalence of attention deficit hyperactivity disorder among children and adolescents in Spain: a systematic review and meta-analysis of epidemiological studies
Source: BMC Psychiatry. 2012 Oct 12;12:168. doi: 10.1186/1471-244X-12-168 (PMC3534011; doi:10.1186/1471-244X-12-168)
Supplement: Additional file 1 — “Search Terms Used In The Bibliographic Review”. [file 1471-244X-12-168-S1.doc]

**WEB APPENDIX**

**ADDITIONAL FILE 1: “SEARCH TERMS USED IN THE BIBLIOGRAPHIC REVIEW”.**

Search Terms Used in Identifying the Epidemiological Studies of ADHD Reporting Prevalence Data (through August 2011)

**MEDLINE (Accessed through PubMed interface from the U.S. National Library of Science):** ((("attention deficit disorder with hyperactivity"[MeSH Terms] OR ("attention"[All Fields] AND "deficit"[All Fields] AND "disorder"[All Fields] AND "hyperactivity"[All Fields]) OR "attention deficit disorder with hyperactivity"[All Fields] OR "adhd"[All Fields]) OR ("attention deficit disorder with hyperactivity"[MeSH Terms] OR ("attention"[All Fields] AND "deficit"[All Fields] AND "disorder"[All Fields] AND "hyperactivity"[All Fields]) OR "attention deficit disorder with hyperactivity"[All Fields]) OR ("hyperkinesis"[MeSH Terms] OR "hyperkinesis"[All Fields] OR "hyperactivity"[All Fields]) OR ("attention deficit disorder with hyperactivity"[MeSH Terms] OR ("attention"[All Fields] AND "deficit"[All Fields] AND "disorder"[All Fields] AND "hyperactivity"[All Fields]) OR "attention deficit disorder with hyperactivity"[All Fields] OR ("attention"[All Fields] AND "deficit"[All Fields]) OR "attention deficit"[All Fields]) OR ("hyperkinesis"[MeSH Terms] OR "hyperkinesis"[All Fields] OR "hyperactivity"[All Fields])) AND ("epidemiology"[Subheading] OR "epidemiology"[All Fields] OR "epidemiology"[MeSH Terms] OR "epidemiology"[Subheading] OR "epidemiology"[All Fields] OR "prevalence"[All Fields] OR "prevalence"[MeSH Terms]) AND (("Spain"[MeSH Terms] OR spain[Text Word]) OR espagne[All Fields] OR espana[All Fields] OR (spain[ad] OR espagne[ad] OR espana[ad]) OR osasunbidea[ad] OR osakidetza[ad] OR insalud[ad] OR sergas[ad] OR (catalunya[ad] OR catalonia[ad] OR catalogne[ad] OR cataluna[ad] OR catala[ad] OR (barcelon[ad] OR barcelona[ad] OR barcelone[ad] OR barcelones[ad] OR barceloneta[ad]) OR tarragona[ad] OR lleida[ad] OR lerida[ad] OR girona[ad] OR gerona[ad] OR sabadell[ad] OR hospitalet[ad] OR l'hospitalet[ad]) OR ((valencia[ad] OR valenciana[ad] OR valenciano[ad]) OR (castello[ad] OR castellon[ad]) OR alacant[ad] OR (alicant[ad] OR alicante[ad]) OR (murcia[ad] OR murcian[ad] OR murciana[ad] OR murciano[ad])) OR (andalucia[ad] OR andaluciajunta[ad] OR andalusia[ad] OR andalusian[ad] OR andaluz[ad] OR andaluza[ad]) OR (sevill[ad] OR sevilla[ad] OR seville[ad]) OR (granada[ad] OR granade[ad]) OR huelva[ad] OR almeria[ad] OR cadiz[ad] OR jaen[ad] OR malaga[ad] OR cordoba[ad] NOT argentina[ad] OR (extremadura[ad] OR caceres[ad] OR badajoz[ad] OR madrid[ad]) OR (castilla[ad] OR salamanca[ad] OR zamora[ad] OR valladolid[ad] OR segovia[ad] OR soria[ad] OR palencia[ad] OR avila[ad] OR burgos[ad]) OR (leon[ad] NOT (france[ad] OR clermont[ad] OR rennes[ad] OR lyon[ad] OR USA[ad] OR (mexic[ad] OR mexica[ad] OR mexican[ad] OR mexicana[ad] OR mexicano[ad] OR mexicanos[ad] OR mexico[ad]))) OR (galicia[ad] OR gallego[ad] OR compostela[ad] OR vigo[ad] OR coruna[ad] OR ferrol[ad] OR orense[ad] OR ourense[ad] OR pontevedra[ad]) OR (oviedo[ad] OR gijon[ad] OR (asturia[ad] OR asturiano[ad] OR asturias[ad] OR asturias'[ad])) OR ((cantabria[ad] OR cantabrico[ad] OR cantabro[ad]) OR santander[ad]) OR (vasco[ad] OR euskadi[ad] OR basque[ad] OR bilbao[ad] OR bilbo[ad] OR (donosti[ad] OR donostia[ad]) OR vizcaya[ad] OR guipuzcoa[ad] OR gipuzkoa[ad] OR alava[ad] OR araba[ad] OR vitoria[ad]) OR ((navarra[ad] OR navarro[ad]) OR pamplona[ad] OR irunea[ad]) OR ((aragon[ad] OR aragones[ad]) OR zaragoza[ad] OR teruel[ad] OR huesca[ad]) OR (mancha[ad] OR ciudad real[ad] OR albacete[ad] OR cuenca[ad]) OR (toledo[ad] NOT (ohio[ad] OR us[ad] OR usa[ad] OR OH[ad])) OR (guadalajara[ad] NOT (mexic[ad] OR mexica[ad] OR mexican[ad] OR mexicana[ad] OR mexicano[ad] OR mexicanos[ad] OR mexico[ad])) OR ((balear[ad] OR balearen[ad] OR baleares[ad] OR balearic[ad] OR balears[ad] OR balearse[ad]) OR mallorca[ad] OR menorca[ad] OR ibiza[ad] OR eivissa[ad]) OR (palmas[ad] OR lanzarote[ad] OR (canaria[ad] OR canarian[ad] OR canarias[ad] OR canario[ad]) OR tenerife[ad])))

***Índice Médico Español* – IME^1^ (Accessed through IME Biomedicine interface from the**

**Spanish Council for Scientific Research [CSIC]):** TDAH OR Trastorno por déficit de atención OR hiperactividad OR hipercinético

***Índice Bibliográfico Español en Ciencias de la Salud* – IBECS^2^ (Accessed through Biblioteca Virtual en Salud [BVS] and Instituto de Salud Carlos III):** TDAH OR Trastorno por déficit de atención OR hiperactividad OR hipercinético

**TESEO^3^ – *Base de datos de Tesis Doctorales* (Accessed through the Spanish Ministry of Education, Culture and Sports):** TDAH OR Trastorno por déficit de atención OR Hiperactividad OR Trastorno hipercinético

*^1^Índice Médico Español* (IME). Free access available at : http://bddoc.csic.es:8080/

^2^*Índice Bibliográfico Español en Ciencias de la Salud* (IBECS). Free access available at: http://ibecs.isciii.es/

^3^*Base de datos de Tesis Doctorales* (TESEO). Free access available at: https://www.educacion.gob.es/teseo/
