# Supplementary material for: Prevalence of attention deficit hyperactivity disorder among children and adolescents in Spain: a systematic review and meta-analysis of epidemiological studies
Source: BMC Psychiatry. 2012 Oct 12;12:168. doi: 10.1186/1471-244X-12-168 (PMC3534011; doi:10.1186/1471-244X-12-168)
Supplement: Additional file 2 — PRISMA Checklist. MOOSE Checklist. [file 1471-244X-12-168-S2.doc]

| **Section/topic** | **#** | **Checklist item** | **Page #** | **Comments** |
| --- | --- | --- | --- | --- |
| **TITLE** | | |  |  |
| Title | 1 | Identify the report as a systematic review, meta-analysis, or both. | 1 |  |
| **ABSTRACT** | | |  |  |
| Structured summary | 2 | Provide a structured summary including, as applicable: background; objectives; data sources; study eligibility criteria, participants, and interventions; study appraisal and synthesis methods; results; limitations; conclusions and implications of key findings; systematic review registration number. | 2 | Observational studies = no registration |
| **INTRODUCTION** | | |  |  |
| Rationale | 3 | Describe the rationale for the review in the context of what is already known. | 3-4 |  |
| Objectives | 4 | Provide an explicit statement of questions being addressed with reference to participants, interventions, comparisons, outcomes, and study design (PICOS). | 4 | Cross-sectional non interventional |
| **METHODS** | | |  |  |
| Protocol and registration | 5 | Indicate if a review protocol exists, if and where it can be accessed (e.g., Web address), and, if available, provide registration information including registration number. | NA |  |
| Eligibility criteria | 6 | Specify study characteristics (e.g., PICOS, length of follow-up) and report characteristics (e.g., years considered, language, publication status) used as criteria for eligibility, giving rationale. | 5-6 | + Tables 1 and 2 |
| Information sources | 7 | Describe all information sources (e.g., databases with dates of coverage, contact with study authors to identify additional studies) in the search and date last searched. | 4-5 | + Webappendix 1 |
| Search | 8 | Present full electronic search strategy for at least one database, including any limits used, such that it could be repeated. | 4-5 | + Webappedix 1 |
| Study selection | 9 | State the process for selecting studies (i.e., screening, eligibility, included in systematic review... | 6 |  |
| Data collection process | 10 | Describe method of data extraction from reports (e.g., piloted forms, independently, in duplicate) and any processes for obtaining and confirming data from investigators. | 6 |  |
| Data items | 11 | List and define all variables for which data were sought (e.g., PICOS, funding sources) and any assumptions and simplifications made. | 5-6 | + Tables 1 and 2 |
| Risk of bias in individual studies | 12 | Describe methods used for assessing risk of bias of individual studies (including specification of whether this was done at the study or outcome level), and how this information is to be used in any data synthesis. | NA | Cochrane risk of bias tool is designed for RCT |
| Summary measures | 13 | State the principal summary measures (e.g., risk ratio, difference in means). | 5 | Prevalence (%) |
| Synthesis of results | 14 | Describe the methods of handling data and combining results of studies, if done, including measures of consistency (e.g., I^2^) for each meta-analysis. | 7 | Cochran’s Q and I² |


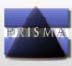
 **PRISMA Checklist**

| **Section/topic** | **#** | **Checklist item** | **Page #** | **Comments** | |
| --- | --- | --- | --- | --- | --- |
| Risk of bias across studies | 15 | Specify any assessment of risk of bias that may affect the cumulative evidence (e.g., publication bias, selective reporting within studies). | 7 | Funnel plot (publication bias) | |
| Additional analyses | 16 | Describe methods of additional analyses (e.g., sensitivity or subgroup analyses, meta-regression), if done, indicating which were pre-specified. | 7 | All of them | |
| **RESULTS** | | | | |  |
| Study selection | 17 | Give numbers of studies screened, assessed for eligibility, and included in the review, with reasons for exclusions at each stage, ideally with a flow diagram. | Figure 1 |  | |
| Study characteristics | 18 | For each study, present characteristics for which data were extracted (e.g., study size, PICOS, follow-up period) and provide the citations. | Tables 1 and 2 |  | |
| Risk of bias within studies | 19 | Present data on risk of bias of each study and, if available, any outcome level assessment (see item 12). | NA |  | |
| Results of individual studies | 20 | For all outcomes considered (benefits or harms), present, for each study: (a) simple summary data for each intervention group (b) effect estimates and confidence intervals, ideally with a forest plot. | 8, 9 | Table 2, Figure 2 | |
| Synthesis of results | 21 | Present the main results of the review. If meta-analyses are done, include for each, confidence intervals and measures of consistency. | 8, 9 |  | |
| Risk of bias across studies | 22 | Present results of any assessment of risk of bias across studies (see Item 15). | NA | Cochrane tool designed for RCT | |
| Additional analysis | 23 | Give results of additional analyses, if done (e.g., sensitivity or subgroup analyses, meta-regression [see Item 16]). | 9, 10, 11 | Webappendix | |
| **DISCUSSION** | | | | |  |
| Summary of evidence | 24 | Summarize the main findings including the strength of evidence for each main outcome; consider their relevance to key groups (e.g., healthcare providers, users, and policy makers). | 11-13 |  | |
| Limitations | 25 | Discuss limitations at study and outcome level (e.g., risk of bias), and at review-level (e.g., incomplete retrieval of identified research, reporting bias). | 12-15 |  | |
| Conclusions | 26 | Provide a general interpretation of the results in the context of other evidence, and implications for future research. | 16 |  | |
| **FUNDING** | | | | |  |
| Funding | 27 | Describe sources of funding for the systematic review and other support (e.g., supply of data); role of funders for the systematic review. | 16 | No funding | |

| **Items** | **Page #** | **Comments** |
| --- | --- | --- |
| **TITLE** Identify the study as a meta-analysis (or systematic review) | 1 |  |
| **ABSTRACT** Use the journal’s structured format | 2 |  |
| **INTRODUCTION** |  |  |
| · The clinical problem | 3,4 |  |
| · The hypothesis | 3 |  |
| · A statement of objectives that includes the study population, the condition of interest, the exposure or intervention, and the outcome(s) considered | 4 |  |
| **SOURCES** |  |  |
| · Qualifications of searchers (eg, librarians and investigators) | 6 | 1 psychiatrist, 1 epidemiologist |
| · Search strategy, including time period included in the synthesis and keywords | 4,5 | Webappendix 1 |
| · Effort to include all available studies, including contact with authors | 5,6 |  |
| · Databases and registries searched | 4,5 | Webappendix 1 |
| · Search software used, name and version, including special features used (eg, explosion) | - |  |
| · Use of hand searching (eg, reference lists of obtained articles) | 5 |  |
| · List of citations located and those excluded, including justification | fig 1 |  |
| · Method of addressing articles published in languages other than English | 5 | IME, IBECS, TESEO |
| · Method of handling abstracts and unpublished studies | 5 | TESEO (thesis) |
| · Description of any contact with authors | - |  |
| **STUDY SELECTION** |  |  |
| · Types of study designs considered | 5,6 | Cross-sectional |
| · Relevance or appropriateness of studies gathered for assessing the hypothesis to be tested | 5,6 |  |
| · Rationale for the selection and coding of data (eg, sound clinical principles or convenience) | 5,6 |  |
| · Documentation of how data were classified and coded (eg, multiple raters, blinding, etc) | 6 |  |
| · Assessment of confounding (eg, comparability of cases and controls in studies where appropriate) | NA |  |
| · Assessment of study quality, including blinding of quality assessors; stratification or regression on possible predictors of study results | 7 | Meta-regressions, subgroup analysis |
| · Assessment of heterogeneity | 7 | Yes |
| · Statistical methods (eg, complete description of fixed or random effects models, justification of whether the chosen models account for predictors of study results, dose-response models, or cumulative meta-analysis) in sufficient detail to be replicated | 7 |  |
| **RESULTS** |  |  |
| · A graph summarizing individual study estimates and the overall estimate | Figure 1 |  |
| · A table giving descriptive information for each included study | Tables 1 and 2 |  |
| · Results of sensitivity testing (eg, subgroup analysis) | 10 | + Webappendix |
| · Indication of statistical uncertainty of findings | 9,10 |  |
| **DISCUSSION** |  |  |
| · Strengths and weaknesses | 11-15 |  |
| · Potential biases in the review process (eg, publication bias) | 14 | Funnel plot (publicacion bias), national registries |
| · Justification for exclusion (eg, exclusion of non–English-language citations) | - |  |
| · Assessment of quality of included studies | - |  |
| · Consideration of alternative explanations for observed results | 15,16 |  |
| · Generalization of the conclusions (ie, appropriate for the data presented and within the domain of the literature review) | 16 |  |
| · Guidelines for future research | 16 | e-g. burden of disease (DALYs) ; cost-effectiveness |
| · Disclosure of funding source | 16 |  |

**MOOSE Checklist**
