# Supplementary material for: Prevalence of attention deficit hyperactivity disorder among children and adolescents in Spain: a systematic review and meta-analysis of epidemiological studies
Source: BMC Psychiatry. 2012 Oct 12;12:168. doi: 10.1186/1471-244X-12-168 (PMC3534011; doi:10.1186/1471-244X-12-168)
Supplement: Additional file 3 — “List Of Excluded References And Reasons for Exclusion”. [file 1471-244X-12-168-S3.doc]

**WEB APPENDIX**

**ADDITIONAL FILE 3: “LIST OF EXCLUDED REFERENCES AND REASONS FOR EXCLUSION”.**

| **References** | **Reason** |
| --- | --- |
| 1. Pedrero-Perez EJ, Ruiz-Sanchez de Leon JM, Rojo-Mota G, et al: Prevalence of attention-deficit hyperactivity disorder in substance addiction: from screening to diagnosis. Rev Neurol. 2011;52(6):331-40. | 1 |
| 2. López-Villalobos JA, Molinero LR, Sacristán Martín AM, et al: Validity of the DSM-IV criteria according to the parents’ response in the diagnosis of the attention deficit hyperactivity disorder. *Rev Asoc Esp Neuropsiq.* 2011;31(110):271-83. | 2 |
| 3. Miranda-Casas A, Presentacion-Herrero MJ, Colomer-Diago C, et al. Satisfaction with life of children with attention deficit hyperactivity disorder: a study of possible protection and risk factors. Rev Neurol. 2011;52 Suppl 1:S119-26. | 3 |
| 4. Aragonès E, Lluís Piñol J, Ramos-Quiroga JA, et al: Prevalence in adults of attention deficit hyperactivity disorder using the medical records of primary care. Rev Esp Salud Publica. 2010;84(4):417-22. | 1 |
| 5. López-Soler C, Castro Sáez M, Alcántara López M, et al: Prevalence and characteristics of externalizing symptoms in childhood. Gender differences. Psicothema. 2009;21(3):353-8. | 4, 5 |
| 6. Haro Cortés G, Benito Delegido A, Ripoll C et al: Attention-deficit hyperactivity disorder in adults with substance use disorders. Trastor. Adict 2009; 11:271-77. | 1, 3 |
| 7. García Vallejo R, García Sanabria S, García Ramos P. Attention Deficit Disorder with or without Hyperactivity. Relationship among Nurses, Parents and School. Rev ROL Enferm 2009; 32:614-20. | 3 |
| 8. Valdizán JR, Izaguerri-Gracia AC. Attention deficit hyperactivity disorder in adults. Rev Neurol. 2009;48 Suppl 2:S95-9. | 1, 6 |
| 9. García García MD, Prieto Tato LM, Santos Borbujo J, et al: Attention deficit and hyperactivity disorder, a current problem. An Pediatr (Barc). 2008;69(3):244-50. | 4 |
| 10. Morán Sánchez I, Navarro-Mateu F, Robles Sánchez F, et al. Evaluation of the validity of AD/HD diagnoses in referrals from paediatrics to the child psychiatry clinic. Aten Primaria. 2008;40(1):29-33. | 3 |
| 11. Tomás Vila M, Miralles Torres A, Beseler Soto B, et al. Attention-deficit/hyperactivity disorder and sleep disturbances. Results of an epidemiological study in schoolchildren in Gandia, Spain. An Pediatr (Barc). 2008;69:251-7. | 3 |
| 12. López-Muñoz F, Alamo C, Quintero-Gutiérrez FJ, García-García P. A bibliometric study of international scientific productivity in attention-deficit hyperactivity disorder covering the period 1980-2005. Eur Child Adolesc Psychiatry. 2008;17(6):381-91. | 3, 6 |
| 13. Pedrero Pérez EJ, Puerta García C. ASRS v.1.1., a tool for attention-deficit/hyperactivity disorder screening in adults treated for addictive behaviors: psychometric properties and estimated prevalence. Adicciones. 2007;19:393-407. | 1, 3 |
| 14. Valdizán JR, Mercado E, Mercado-Undanivia A. Clinical variability and characteristics of attention deficit hyperactivity disorder in girls. Rev Neurol. 2007;44 Suppl 2:S27-30. | 4, 5 |
| 15. Servera M, Cardo E. ADHD Rating Scale-IV in a sample of Spanish schoolchildren: normative data and internal consistency for teachers and parents. Rev Neurol. 2007 Oct 1-15;45(7):393-9. | 3 |
| 16. Poeta LS, Rosa-Neto F. Motor assessment in school-aged children with indicators of the attention deficit/hyperactivity disorder. Rev Neurol. 2007;44:146-9. | 3, 7 |
| 17. Rojo L, Ruiz E, Domínguez LA, et al. Comorbidity between obesity and attention deficit/hyperactivity disorder: population study with 13-15 year-olds. Int J Eat Disord 2006; 39:519–522. | 1, 3 |
| 18. Canals Baeza A, Romero Escobar H, Cantó Oíez T et al: Inclusion of a screening scale for attention deficit hyperactivity disorder in a primary care program. Acta Pediatr. Esp 2006;64(3):99-102. | 3 |
| 19. Poeta LS, Rosa-Neto F. The biopsychosocial characteristics of schoolchildren with attention deficit hyperactivity disorder indicators. Rev Neurol. 2006;43(10):584-8. | 3, 7 |
| 20. Amador-Campos JA, Forns-Santacana M, Guàrdia-Olmos J, et al. DSM-IV Attention Deficit Hyperactivity Disorder symptoms: agreement between informants in prevalence and factor structure at different ages. J Psychopathol Behav Assess. 2006;28:21-32. | 5 |
| 21. García Lara GA. Trastorno por déficit de atención e hiperactivad y problemas escolares asociados en alumnos de una zona rural e indígena de Chiapas. Rev Psicopatol Salud Ment Niño Adolesc; 2006:155-66 | 7 |
| 22. Fernández Pérez M, López Benito MM: Trastorno por déficit de atención con o sin hiperactividad: evaluación en la consulta pediátrica. Pediatr Aten Prim. 2006; 8(supl. 4):11-24. | 6 |
| 23. Cornejo JW, Osío O, Sánchez Y, et al: Prevalence of attention deficit hyperactivity disorder in Colombian children and teenagers. Rev Neurol. 2005;40(12):716-22. | 7 |
| 24. Amador-Campos JA, Forns-Santacana M, Martorell-Balanzó B, et al. Confirmatory factor analysis of parents' and teachers' ratings of DSM-IV symptoms of attention deficit hyperactivity disorder in a Spanish sample. Psychol Rep. 2005;97:847-60. | 3 |
| 25. Criado Alvarez JJ, Romo Barrientos C. Variability and tendencies in the consumption of methylphenidate in Spain. An estimation of the prevalence of attention deficit hyperactivity disorder. Rev Neurol. 2003;37:806-10. | 5 |
| 26. Montiel-Nava C, Peña JA, Montiel-Barbero I. Epidemiological data about attention deficit hyperactivity disorder in a sample of Marabino children. Rev Neurol. 2003;37(9):815-9. | 7 |
| 27. Eddy LS, Toro Trallero J, Salamero Baró M, et al. Attention deficit hyperactivity disorder. A survey to evaluate risk factors, associated factors and parental child rearing behavior. An Esp Pediatr.1999;50:145-50. | 2 |
| 28. Eddy LS. Estudio del trastorno por déficit de atención con hiperactividad. Prevalencia, factores de riesgo y factores asociados. *PhD thesis*. University of Barcelona; 1997. | 2 |
| 29. García-Álvarez R, Quintero Lumbreras FJ, Herrera Pino JA, et al: Prevalencia del síndrome de déficit de atención, hiperactividad e impulsividad. Psiquis 1996;17:369-382. | 7 |
| 30. Trillo M: Síndrome hipercinético en adolescentes. Arch Pediatr. 1996;47:55-60. | 6 |
| 31. Benjumea Pino P, Mojarro Praxedes MD, Peiró S: Comorbilidad, ansiedad y trastornos hipercinéticos y de conducta. An Psiquiatr 1996;12:290-94. | 3 |
| 34. Catalá Angel MA, Andrés Carrasco MA, Gómez Beneyot M, Agüero Juan A. Validez actual de los criterios del Barkley para el diagnóstico del trastorno por deficit de atención con hiperactividad. Rev Psiquiatr Infant Juv 1994;3:198-202. | 3 |
| 33. Andrés Carrasco MA. Estudio de prevalencia del trastorno por déficit de atención con hiperactividad en niños de 10 años residentes en un municipio de Valencia. *PhD thesis*. University of Valencia; 1993. | 2 |
| 34. Avila de Encio MC, Polaina Lorente A. Adaptación de la entrevista parental account of childhood symptoms en la población escolar para la evaluación de la hiperactividad infantil. Rev Psiquiatr Infant Juv 1992;4:241-50. | 3 |
| 35. León Carrión J, Valencia Pino JA. Perspectiva comprensiva de los aspectos neuropsicológicos en niños con síndrome por déficit de atención con hiperactividad a través de los resultados obtenidos en el test de Roschach. Psiquis 1986;7:57-64 | 3 |

Reasons for exclusion: 1. Study population; 2. Duplicate; 3. Not relevant topic; 4. Clinical sample; 5. Does not report outcome of interest; 6. Review/commentary; 7. Not conducted in Spain
