# Supplementary material for: Prevalence of attention deficit hyperactivity disorder among children and adolescents in Spain: a systematic review and meta-analysis of epidemiological studies
Source: BMC Psychiatry. 2012 Oct 12;12:168. doi: 10.1186/1471-244X-12-168 (PMC3534011; doi:10.1186/1471-244X-12-168)
Supplement: Additional file 4 — “Uncertainty And Sensitivity Analyses”. [file 1471-244X-12-168-S4.doc]

**WEB APPENDIX**

**ADDITIONAL FILE 4: “UNCERTAINTY AND SENSITIVITY ANALYSES”.**

| **Figure S-1: Suplementary meta-analysis of the prevalence of ADHD among children and adolescents in Spain: exclusion of outliers.** |
| --- |
|  |
| Note: Random-effects model.  Cochran’s Q: χ^2^ = 28.06 (d.f. = 11), p=0.003; I^2^=60.8%. Excluding 4 outlier estimates [references 2, 5, 15 and 18], the pooled prevalence was 5.3% (95% 4.5-6.2%) representing 281,819 (95% CI 239,281 – 329,676) children and adolescents in the community. |

**WEB APPENDIX**

**ADDITIONAL FILE 4: “UNCERTAINTY AND SENSITIVITY ANALYSES”.**

| **Figure S-2: Suplementary meta-analysis of the prevalence of ADHD among children and adolescents in Spain: fixed-effects model.** |
| --- |
|  |
| Note: Fixed-effects model.  Cochran’s Q: χ^2^ = 367.7 (d.f. = 15), p=0.001; I^2^=95.9%. The pooled prevalence was 5.1% (95% 4.7-5.5%) representing 271,185 (95% CI 249,915 – 292,454) children and adolescents in the community. |

**WEB APPENDIX**

**ADDITIONAL FILE 4: “UNCERTAINTY AND SENSITIVITY ANALYSES”.**

| **Figure S-3: Suplementary meta-analysis of the prevalence of ADHD among children and adolescents in Spain: on the basis of clinical ascertainment (clinically confirmed).** |
| --- |
|  |
| Note: Random-effects model.  Cochran’s Q: χ^2^ = 44.87 (d.f. = 8), p<0.001; I^2^=82.2%. The pooled prevalence was 6.4% (95% 4.9-7.9%) representing 340,310 (95% CI 260,550 – 420,070) children and adolescents in the community. |
